# Low-Dose Quetiapine in the Treatment of SSRI-Induced Bruxism and Mandibular Dystonia: Case Series

**Published:** 2018-07

**Authors:** Atefeh Zandifar, Mohammad Reza Mohammadi, Rahim Badrfam

**Affiliations:** 1 Imam Hossein Hospital, Alborz University of Medicine Sciences, Karaj, Iran.; 2 Psychiatry and Psychology Research Center, Roozbeh Hospital, Tehran University of Medicine Sciences, Tehran, Iran.; 3 Roozbeh Hospital, Tehran University of Medicine Sciences, Tehran, Iran.

**Keywords:** *Bruxism*, *Mandibular Dystonia*, *Quetiapine*, *Serotonin Reuptake Inhibitors (SSRIs)*

## Abstract

**Objective:** Selective serotonin reuptake inhibitors (SSRIs) have been the most widely used psychopharmacological agents prescribed for depression worldwide. Some adverse effects of SSRI drugs on central nervous system are insomnia and bruxism. These drugs also affect sleep. Quetiapine is used as adjunctive therapy to antidepressants for the treatment of major depressive disorder (MDD). It is a low- dose dibenzothiazepine with more potent 5-HT2 than D2 receptor-blocking properties that can be used to manage bruxism because of its antagonist effect on the 5-HT2 receptor.

**Cases:** The cases were 5 patients who have recently been treated with SSRIs and presented with bruxism. Low- dose quetiapine (between 25 and 50 mg daily) was prescribed for the patients, and after a few days, they reported no bruxism and continued taking the medication.

**Conclusion: **We found that quetiapine can improve bruxism and mandibular dystonia, which are side effects of SSRIs.

SSRIs are the most widely prescribed psychopharmacological agents for depression worldwide. They are approved by the FDA for the following indications: major depressive disorder (MDD), obsessive compulsive disorder (OCD), posttraumatic stress disorder (PTSD), panic disorder, and social phobia. SSRIs are clearly superior to older antidepressant medications in safety (1). Considering the side effects, SSRIs have been found to be more problematic than the suggested original clinical trials. However, they may rarely cause extrapyramidal symptoms such as akathisia, dystonia, and tremor. Nevertheless, some adverse effects of SSRI drugs on central nervous system are insomnia and bruxism; these drugs may also cause sleep disorders such as vivid dreams and restless legs (2-6).

Many clinical trials have been conducted to reduce these side effects. In particular, many studies aimed at determining the effects of some drugs such as buspirone (7, 8, 9 and 10-13) , aripiprazole (14), trazodone (15), and gabapentin on treating bruxism and jaw dystonia (16, 17). Another study was done to assess the effects of quetiapine on controlling tremulous jaw movements in rats (18) and found that quetiapine can successfully manage tardive dyskinesia (19). Masticatory motor activity is controlled by dopaminergic neurons of the mesocortical tract (14). 5-HT1 and 5-HT3 receptor agonists facilitate dopamine release while agonists on 5-HT2 receptors inhibit dopamine release (11, 12 and 13).

 Moreover, 5-HT2 blockade would theoretically result in less inhibition of the dopaminergic neurons and, hence, less interference with mesocortical tract dopamine release.Quetiapine is a dibenzothiazepine with more potent 5-HT2 than D2 receptor-blocking properties (It has high affinity for 5-HT2, a moderate affinity for 5-HT1A and D2 receptors, and a low affinity for D1 receptors). 

Low incidence of EPS in quetiapine (for example akathisia) may be explained by its relatively high 5-HT2A binding compared to its activity at D2 receptors (1).

Quetiapine is used to treat schizophrenia, is indicated as monotherapy for the acute treatment of depressive episode associated with bipolar disorder, and it is also used as adjunctive therapy to antidepressants for the treatment of MDD(2).

The clinical effects of quetiapine and also its effects on the mentioned receptors encouraged us to provide a report on a number of our patients who were suffering from side effects of bruxism due to the use of SSRIs. However, their condition improved after using low- dose quetiapine.

## Case Reports

**CASE 1:** the first case was a 28- year-old female with MDD (depressed mood, weight loss, and psychomotor retardation, loss of energy, and insomnia for more than 1 month). She was started on 75mg sertraline daily but started to suffer from bruxism and jaw spasm in the second week of receiving the medication. She stopped taking sertraline because of its side effects and immediately visited a psychiatrist because of her depression and bruxism. Again, she was prescribed 75mg sertraline and 25mg quetiapine. Her bruxism improved after 5 days, and she remained symptom- free throughout the following month.

**CASE 2:** the second case was a 35- year-old male with episodes of MDD during last 5 years and suffered some side effects, such as akathisia, restlessness, bruxism and mandibular dystonia, after taking sertraline. He discontinued the medication due to the side effects. Then, he was prescribed sertraline 12.5 mg daily and the dose was increased to 50 mg daily. However, after a week, he began to suffer from bruxism and mandibular dystonia. Thus, he started taking quetiapine (25 mg one time at night) and his condition improved after 5 days and continued taking SSRI drug for his major depressive disorder.

**CASE 3:** the third case was an 18-year-old female with MDD during last 2 months, with depressed mood, restlessness, diminished ability to concentrate, fatigue, and insomnia. She was prescribed with fluoxetine (20 mg daily), but shortly after the onset of drug treatment, she developed bruxism. She improved significantly after one week by taking quetiapine 12.5 mg in the morning and 25 mg at night, and she completely improved after 3 weeks.

**CASE 4: **Our fourth case was a 45-year-old female with MDD (depressed mood, significant weight loss, insomnia, fatigue, and feeling of worthlessness, diminished interest in all activities most of the day for more than 1 month). She started sertraline 12.5 mg daily and had good drug compliance. Three weeks after sertraline dose was increased to 50 mg daily, she developed bruxism, lip movements, and jaw dystonia. She received quetiapine (12.5 mg in the morning and 25 mg at night) and, as a result, her condition improved after 5 days, and she remained symptom-free throughout the following month. 

**CASE 5:** Our fifth case was a 32-year-old female with postpartum depression 5 years ago. She had been admitted to the hospital due to suicidal ideas at that time. She received ECT and citalopram, clonazepam, and quetiapine, and her condition improved completely. She had no psychiatric problems for the last 5 years up to 2 months ago when she showed restlessness, sleep problems, depressed mood, decreased social function, and suicidal ideas. We recommended hospitalization because of her MDD diagnosis, but she disagreed, so we started her on citalopram (10 mg daily) and clonazepam (1 mg daily) in an outpatient setting. Two weeks after starting the medication, she showed partial improvement. The patient suffered from bruxism and, thus, was prescribed quetiapine (25 mg daily); and 10 days after taking quetiapine, she reported no bruxism and continued taking citalopram**.**

## Discussion

In these case series, we reported 5 patients with MDD who received SSRIs medication. They suffered from some side effects including bruxism, jaw dystonia, restlessness, akathisia, and insomnia due to taking antidepressant medication. Some of the patients stopped taking their medication and suffered relapse or continuance of symptoms and some others returned to their psychiatrics because of side effects.

 To treat bruxism and mandibular dystonia due to SSRIs medication, we used low- dose quetiapine (between 25 and 50 mg daily) and observed improvement in these side effects, which permitted the continuance of SSRIs medication to treat psychiatric diseases. The effectiveness of low- dose quetiapine in managing bruxism, as the beneficial effect of trazodone on bruxism (15), may be due to its antagonist effect on the 5-HT2 receptor.

Thus, low- dose quetiapine should be used to improve bruxism and mandibular dystonia as side effects of medications with SSRIs due to their high affinity to 5-HT2 receptor and their usefulness as an adjunctive therapy to antidepressants to treat MDD(2). Moreover, low- dose quetiapine (between 12.5 mg and 100 mg daily) should be prescribed to treat insomnia, anxiety, and agitation because of its sedative and somnolent properties (1, 20) (We also observed this ability of low- dose quetiapine in our cases).

Future studies should be conducted to examine the effect of quetiapine on improving the mentioned complications in larger groups of patients with SSRIs side effects.

## Conclusion

Based on the results of the present study, low- dose quetiapine can improve bruxism and mandibular dystonia, which are side effects of SSRIs. This led to possibility of continuing treatment with SSRIs to control patients’ disorders.
